# Septum Development in *Neurospora crassa*: The Septal Actomyosin Tangle

**DOI:** 10.1371/journal.pone.0096744

**Published:** 2014-05-06

**Authors:** Diego Luis Delgado-Álvarez, Salomón Bartnicki-García, Stephan Seiler, Rosa Reyna Mouriño-Pérez

**Affiliations:** 1 Departamento de Microbiología, Centro de Educación Científica y de Educación Superior de Ensenada, Ensenada, B. C. México; 2 Institute for Biology II – Molecular Plant Physiology, Albert-Ludwigs University Freiburg, Freiburg, Germany; 3 Freiburg Institute for Advanced Studies (FRIAS), Albert-Ludwigs University Freiburg, Freiburg, Germany; University of Nebraska, United States of America

## Abstract

Septum formation in *Neurospora crassa* was studied by fluorescent tagging of actin, myosin, tropomyosin, formin, fimbrin, BUD-4, and CHS-1. In chronological order, we recognized three septum development stages: 1) septal actomyosin tangle (SAT) assembly, 2) contractile actomyosin ring (CAR) formation, 3) CAR constriction together with plasma membrane ingrowth and cell wall construction. Septation began with the assembly of a conspicuous tangle of cortical actin cables (SAT) in the septation site >5 min before plasma membrane ingrowth. Tropomyosin and myosin were detected as components of the SAT from the outset. The SAT gradually condensed to form a proto-CAR that preceded CAR formation. During septum development, the contractile actomyosin ring remained associated with the advancing edge of the septum. Formin and BUD-4 were recruited during the transition from SAT to CAR and CHS-1 appeared two min before CAR constriction. Actin patches containing fimbrin were observed surrounding the ingrowing septum, an indication of endocytic activity. Although the trigger of SAT assembly remains unclear, the regularity of septation both in space and time gives us reason to believe that the initiation of the septation process is integrated with the mechanisms that control both the cell cycle and the overall growth of hyphae, despite the asynchronous nature of mitosis in *N. crassa*.

## Introduction

Filamentous fungi such as the ascomycete model fungi, *Neurospora crassa* and *Aspergillus nidulans,* grow by apical extension of tubular cells called hyphae. These cells are compartmentalized by cross walls that retain a central pore through which cytoplasm and organelles including nuclei flow freely. Although the precise function of the septum has not been well established, some explanations have been proposed, such as providing structural integrity to the hyphal tube, preventing cytoplasmic leakage by plugging the septal pore with Woronin bodies, and participating in cell differentiation [1]–[6]. Hyphal septa are not essential, since groups of fungi do well without them. However, mutants of *N. crassa* lacking septa display cytoplasmic leakage and are deficient in conidiation [7]–[10].

Hyphal septation and yeast cytokinesis are alike but without the final step of cell separation. These two processes share key features such as the selection of the cross wall formation site, the assembly of a contractile actomyosin ring (CAR) and the coupled processes of plasma membrane ingrowth and cell wall construction [11]–[16]. In contrast to unicellular yeasts, not every nuclear division is coupled with cytokinesis in filamentous fungi, resulting in the formation of multinucleated hyphal compartments. Thus, nuclear position and cell cycle seem only loosely coordinated with septum genesis. Nevertheless, CAR assembly and septum formation are clearly controlled through nuclear position and cell cycle progression in *A. nidulans* [17]–[21]. Although a similar correlation between mitosis and septation may also apply to *N. crassa* [22]–[26], the asynchronous mitosis in this fungus makes the connection between cell cycle and septum formation difficult to establish.

Hyphae are compartmentalized with precision, suggesting that septum initiation is a well-regulated event [21]. However, the mechanism for determining the site of septum formation is one of the least-conserved aspects in fungi. Budding and fission yeast, for example, have developed fundamentally distinct mechanisms to ensure proper nuclear segregation. The site of bud emergence in *Saccharomyces cerevisiae* uses cortical cues from the previous cell division cycle, while in *Schizosaccharomyces pombe*, opposing nuclear and cell end-dependent spatial signals are integrated in order to trigger medial cell division [14], [27]. However, in both cases cortical anillin-like landmark proteins and a highly conserved kinase cascade called the septation initiation network (SIN) are critical for selection of the division site, temporal-spatial organization of the CAR and coordination of cell cycle progression with CAR constriction [28]. The homologs of the *S. cerevisiae* axial bud site marker proteins Bud3p and Bud4p are important for septum formation in *N. crassa* and *A. nidulans* [8], [29]–[30]. In *N. crassa*, both proteins appear prior to the formation of a detectable septum as cortical rings at incipient septation sites that contract with the forming septum [8].

By detailed characterization of the time of appearance of key proteins involved in septum formation, we were able to construct the chronology of events during septum development in *N. crassa*. Our observations indicate that the first evidence of septum initiation is the assembly of a broad tangle of long actomyosin cables, the septal actomyosin tangle (SAT); this SAT structure becomes compacted to form a tight ring, the CAR. We determined that, surprisingly, BNI-1, the only formin in *N. crassa*, and the landmark protein BUD-4 enter the septation process only at later stages during the transition of the SAT to a mature CAR.

## Materials and Methods

### Strains and Culture Conditions

Strains used in this study are listed in Table 1. Strains were maintained on Vogel’s minimal medium (VMM) with 2% sucrose and 1% agar at room temperature and at 30°C [31].

**Table 1:** *Neurospora crassa* strains used in this study.

Strain	Genotype	Reference
BNI-1-GFP	*bni-1Δ::hph his-3^+^::Pccg-1-bni-1-sgfp+*	[8]
BUD-4-GFP	*bud-4Δ::hph his-3^+^::Pccg-1-bud-4-sgfp+*	[8]
CHS-1-GFP	*mat A his-3+::Pccg-1::chs-1::sgfp+*	[38]
FIM-1-GFP	*mat a his-3^+^::Pccg-1-fim-1 (abd2)-sgfp+*	[35]
Lifeact-GFP	*mat a his-3^+^::Pccg-1-lifeact-egfp+*	[35]
MYO-2-GFP	*Pmyo-2::myo-2-gfp+*	[73]
TPM-1-GFP	*mat a his-3^+^::Pccg-1-tpm-1-sgfp+*	[35]

### Laser Scanning Confocal Microscopy of Living Cells

Strains containing *fim-1::sgfp*, *myo-2::gfp tpm-1::sgfp*, *bud-4::sgfp*, *bni-1::sgfp*, *chs-1::sgfp* and *Lifeact::egfp* were grown on VMM. The “inverted agar block” method [32] was used for live-cell imaging with an inverted laser scanning microscope (LSM-510 Meta, Carl Zeiss, Göttingen, Germany) equipped with an argon ion laser for excitation at 488 nm wavelength and GFP filters for emission at 515–530 nm and 600–700 nm filter for FM4-64. Oil immersion objectives were used: 100× (PH3)/1.3 N. A., 63× (DIC)/1.4 N. A. and 20× (PH2)/0.75 N. A. Laser intensity was kept to a minimum (1.5%) to reduce photobleaching and phototoxic effects [33]. Time-lapse imaging was performed at scan intervals of 0.5 to 13 s for periods up to 60 min. Image resolution was 512×512 pixels and 300 dpi. Confocal images were captured using LSM-510 software (version 3.2; Carl Zeiss) and evaluated with an LSM 510 Image Examiner. Some of the image series were converted into movies (*.mpeg and *.avi) using AVS Video Converter v8.1 (Online Media Technologies LTD.). Final images were processed, and figures were created using Adobe Photoshop CS5 Extended (Adobe Systems Inc., San Jose, CA).

### Membrane Fluorescent Staining

Using the “inverted agar block method” of preparing and staining samples [32], GFP-expressing strains were incubated with 10 µl of 2.5 µM FM4-64 (Molecular Probes, Eugene, OR), which labels the plasma membrane and organelle membranes [34].

For time measurements, we defined time 0∶00 (min: sec) as the moment at which there is first evidence of plasma membrane ingrowth as revealed by FM4-64 staining. Negative time values indicate events occurring prior to membrane ingrowth.

## Results

### Septa are Formed at Regular Intervals in Mature Leading *N. crassa* Hyphae

**Figure 1:**
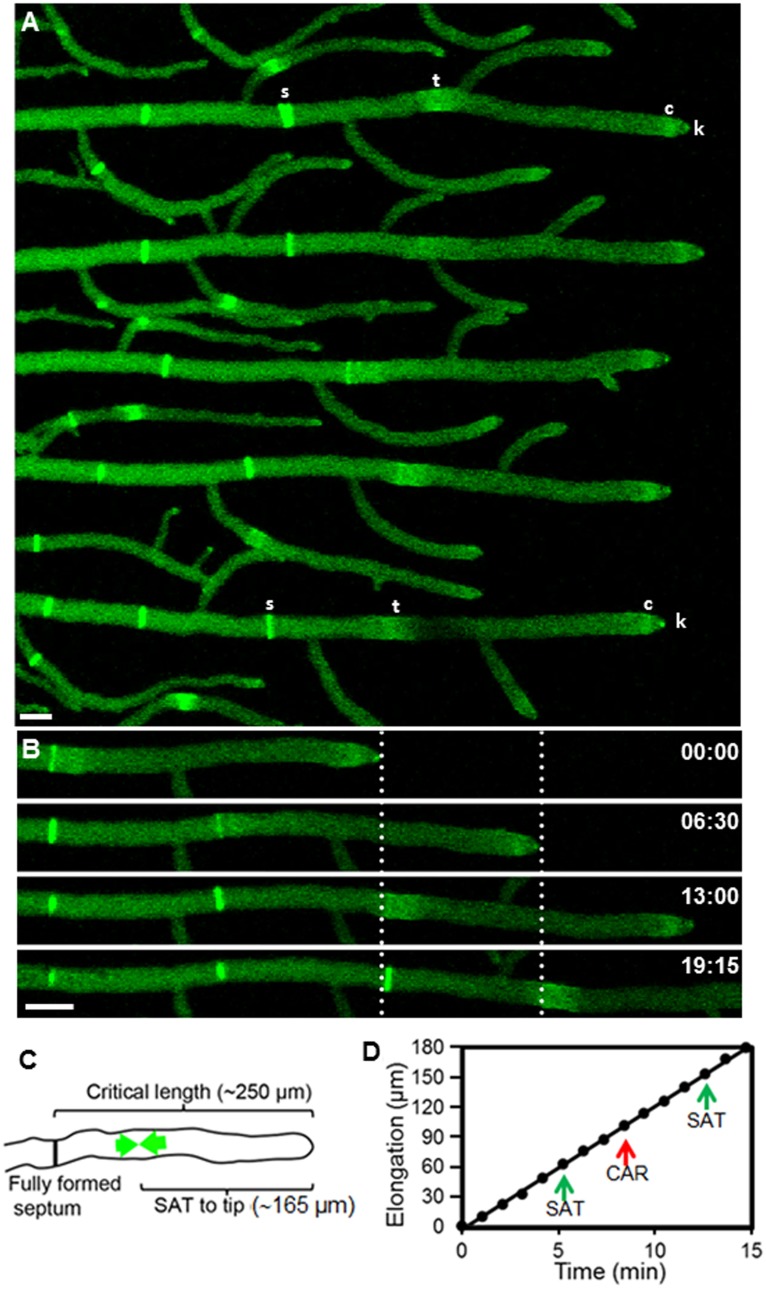
Septum development in hyphae of *Neurospora crassa* visualized by fluorescent tagging of actin with Lifeact-GFP. (A) Growth of primary hyphae monitored for up to 20 min. Lifeact-GFP fluorescence reveals the presence of actin in septa (s) and septal actomyosin tangles (t). Actin is also present in subapical collars (c) and the Spitzenkörper (k). (B) Stages in the septation of a hypha. The dotted lines mark the position of the tip at two consecutive times and, predictably, the place where septation occurred ∼6 min later. (C) Critical hyphal dimensions for septation. As a hypha reaches a critical length of ∼250 µm from the last septation site, a new SAT begins to assemble (green arrows) at the future septation site located at about 165 µm from the tip. (D) Kinetics of hyphal elongation and timing of two SAT and CAR events. Note septum formation did not affect the apical growth rate. Scale Bar = 10 µm.

Septum formation occurred at more or less regular intervals (93±2 µm, n = 36) (mean ± standard error) along the hyphal tube (Fig. 1A, Movie S1). When the apical compartment (the segment between the tip and the nearest septum) reached a critical length (250±5 µm; n = 10) a new septum started to develop. Figure 1B shows the regularity of septation in both space and time. The distance from the apex to the new septation site was 165±3 µm (n = 50); septation splits the leading hyphal compartment into two unequal segments, the apical one being about 2.5 × longer (Fig. 1C). Septum formation occurred without affecting the apical growth rate (13 µm min^−1^) (Fig. 1D). Above measurements were made from time-series of *N. crassa* tagged with Lifeact-GFP (Movie S1).

### The Initial Step in Septation: SAT Formation

**Figure 2:**
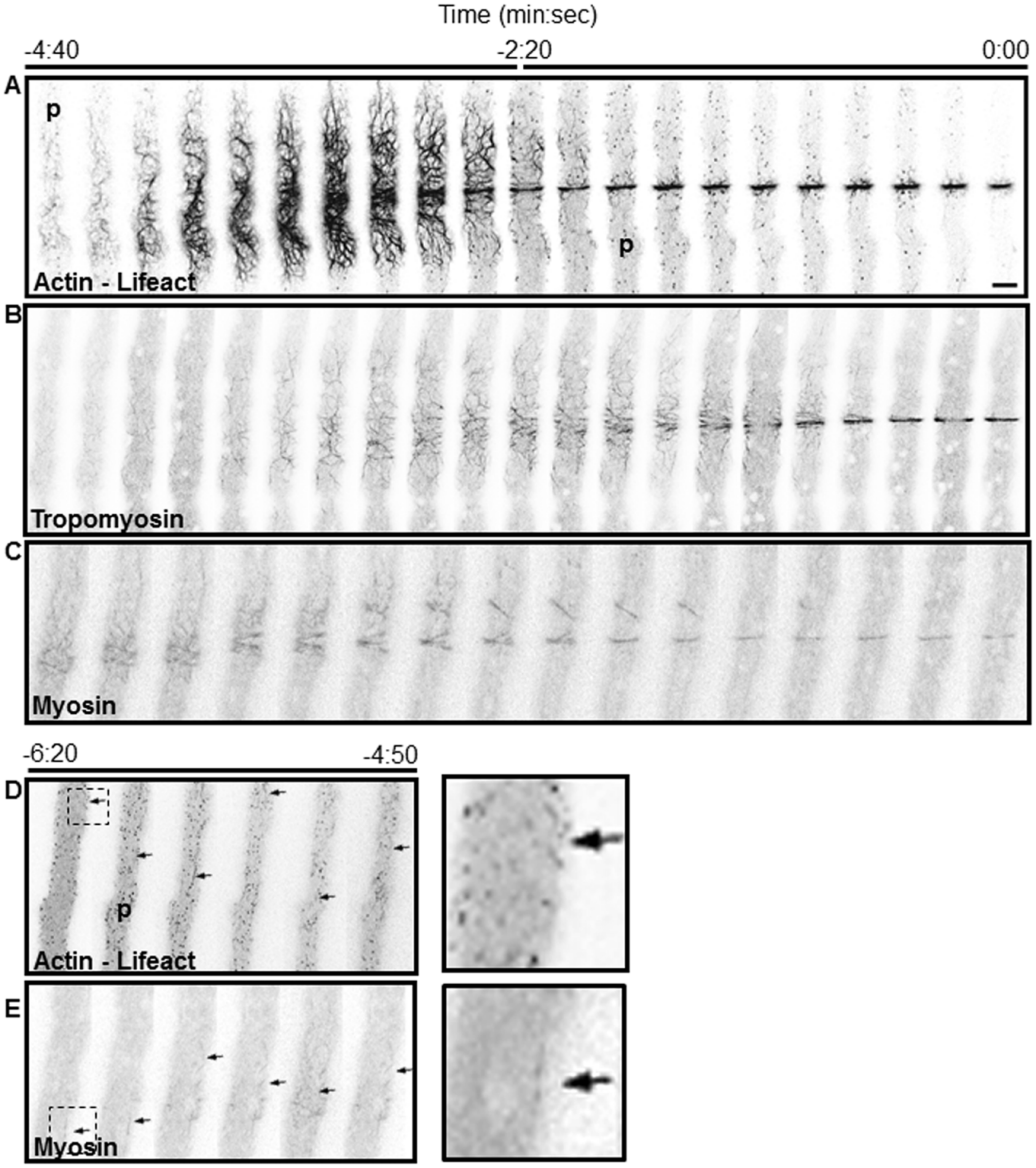
Dynamics of SAT formation and its transition to CAR. Each panel is a series of images taken at 15(SAT): actin (A and D), tropomyosin (B) and MYO-2 (C and E) and the conversion of SAT to a constriction ring (CAR). Actin, tropomyosin and myosin were imaged near the hyphal cell cortex in the correspondingly tagged strains. Time 0∶00 signals the earliest moment of membrane invagination. SAT assembly reached a peak at −3∶00 min. Note that actin patches (p) in panel A are absent during the SAT stage and begin to appear during CAR formation from time −4∶40 to −2∶20 min. The helicoidal actin cables coalesce to from a proto CAR that is finally assembled into a CAR at approximately −2∶30 min (see Movie S2). Panels (D) and (E) show extended time for actin and myosin, arrows point to the incipient formation of short filaments. The panels on the right in (D) and (E) are enlargements of the areas marked at time −6∶20 min. Scale Bar = 5 µm.

**Figure 3:**
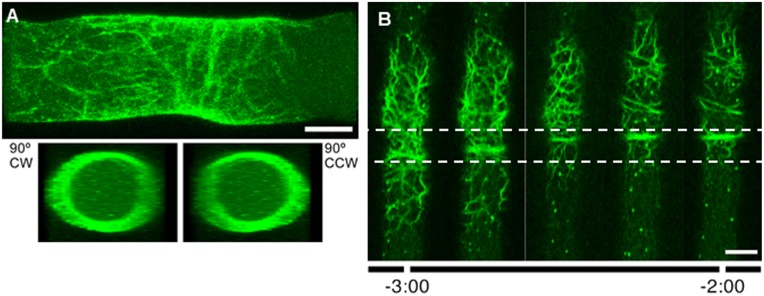
Structure of the septal actomyosin tangle (SAT) labeled with Lifeact-GFP. (A) 3D reconstruction of a z-stack of 0.9 µm confocal sections of a SAT. The upper panel shows a longitudinal view of the 3D reconstruction and the lower panels are transverse views of the rotated reconstruction from both sides. The tangle is a network of roughly helicoid actin cables located in the cell cortex (see also Movie S3) (B) Details of the assembly of an incipient ring (proto-CAR) and its migration as it becomes a mature CAR. Dotted lines frame the zone of displacement of the ring (4 µm for this particular hypha). Scale Bar = 5 µm.

The earliest indicator of septum development was the assembly of a conspicuous tangle of actin filaments imaged by Lifeact-GFP near the site where a new septum will be formed (Fig. 2; Movie S2). Tropomyosin and class II myosin were found associated with the actin cables. We named this complex network of cables the Septal Actomyosin Tangle (SAT). The first evidence of a SAT was the appearance of long actin filaments in the cortical region of the hyphal apical compartment; the filaments exhibited a marked helical tendency and a longitudinal orientation (Figs. 2, 3). The filaments increased in number and thickness progressively forming a cortical meshwork of actomyosin cables. The SAT covered an average length of 35 µm and its formation took place at about 165 µm from the apex. Although the first indication of fine dispersed actin cables was detected at −6∶20 min∶sec, the SAT formation occurred at −4∶30±0∶30 min∶sec (n = 18) (Fig. 2D–E, arrows). The localization of the SAT cables was at the cell cortex, as demonstrated by a three-dimensional reconstruction (Fig. 3A; Movie S3).

### Transition from SAT to CAR

The SAT reached its maximum expression in both number and thickness of filaments at about −3∶00 min (n = 18) (Fig. 2A). The collection of extended helicoidal cables began to compress longitudinally towards a mid-point in the tangle. Compaction of the SAT was not symmetrical; the anterior side of the tangle seemed to contribute more cables to the CAR than the posterior side. The first evidence of a ring appeared at about −2∶48 min∶sec (n = 18). We referred to this structure as a proto-CAR to emphasize that this ring structure had reached neither final place nor size. Cable condensation continued as the proto-CAR migrated forward a distance of ∼4 µm until it became the final CAR (Fig. 3B). The CAR was assembled at −2∶00 min (n = 18), but its constriction started at zero time (Fig. 2A). The complete process of SAT and CAR formation is a spectacular event that lasted ∼7 minutes (n = 18); it can be seen in speeded up fashion in Movie S2 (Fig. 2A). The next phase, from plasma membrane ingrowth to septum completion, took approximately 5∶30 min.

**Figure 4:**
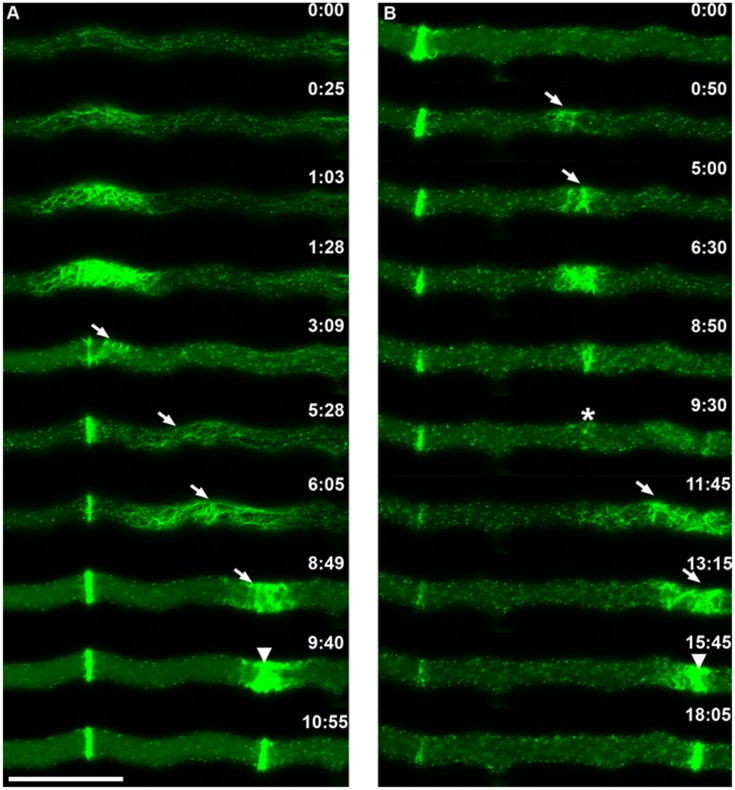
Details of SAT and CAR assembly during septation and an instance of CAR abortion. (A) Normal septation. Actin cables emanate from a recently formed septum and assemble a new SAT that moves towards (arrows) the next septation site where it coalesces to form a CAR (arrowhead) (B) CAR abortion. In this instance, a SAT began to be established at the expected site (arrows); by 6∶30 min it had reached a maximum size which was much smaller than a normal SAT and by 9∶30 min it had almost disappeared (asterisk); no septum was formed, instead the actin cables from the remains of the aborted CAR migrated towards a new site (arrowhead). This time the SAT proceeded to form a normal CAR (arrowhead). Scale Bar = 10 µm.

**Figure 5:**
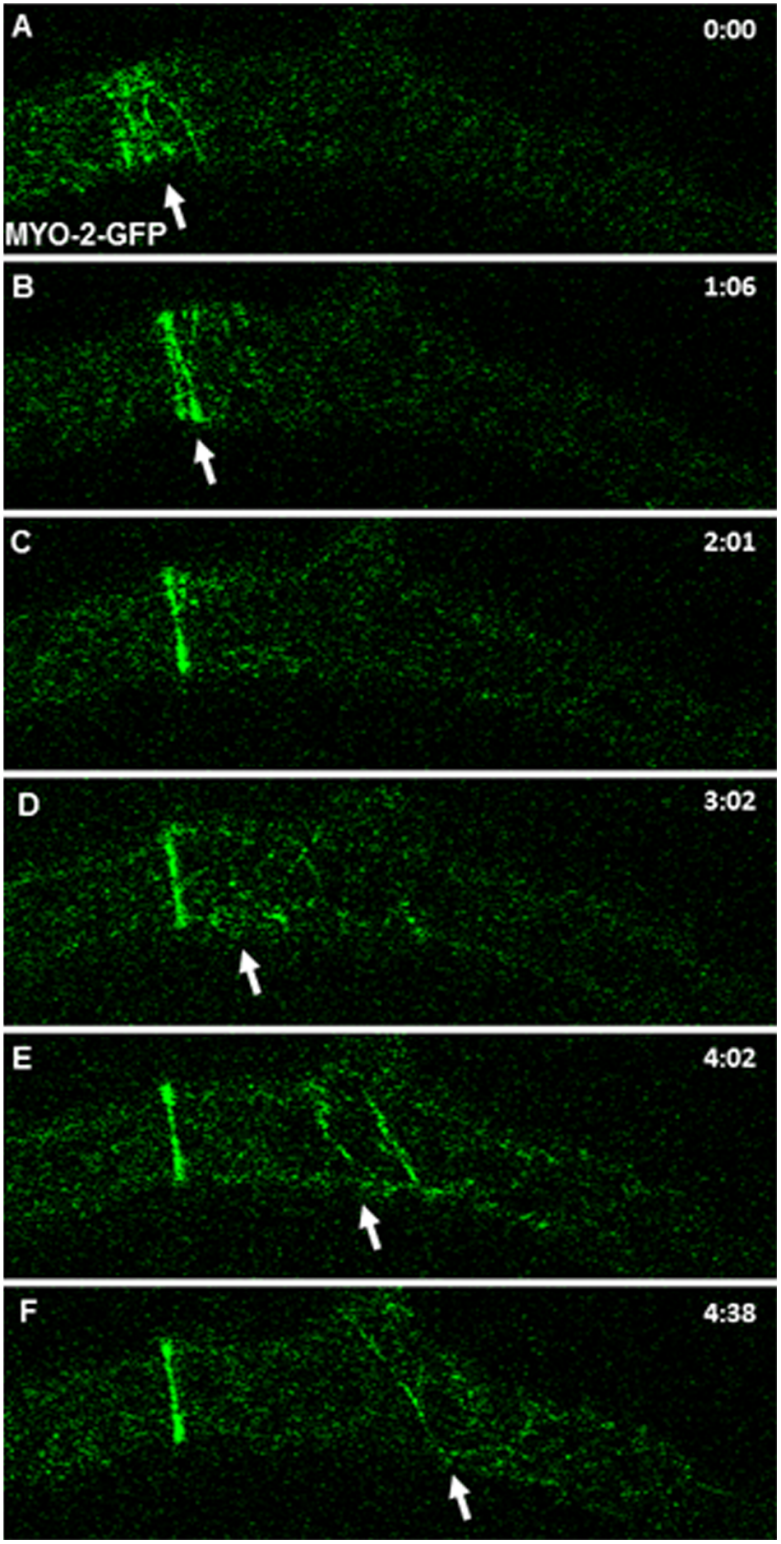
Dynamics of MYO-2-GFP filaments during SAT and CAR assembly. MYO-2 filaments derived from a recently formed septum moved towards (arrows) the next septation site.

SAT formation was a discrete event in what seemed to be a tenuous cloud of Lifeact-GFP fluorescence that traveled in tipward direction from a formed septum to the next septation site (Fig. 4A). The pulse of fluorescence advanced from the proximal edge of the current SAT to the next septation site at 0.4 µm s^−1^ (n = 5), twice the hyphal growth rate (See Movie S4). Within the cloud of fluorescence, it was possible to visualize some small actin cables that traveled from the forming SAT to the next septation site (Fig. 4A). A similar displacement was noticed for MYO-2-GFP. Following the condensation of the SAT into a CAR, part of the MYO-2-GFP filaments traveled toward the next septation site (Fig. 5 and Movie S5).

An unexpected event was the occasional abortion of the CAR formation process (Fig. 4B). A SAT began to assemble in the expected site but never fully developed; after it started to coalesce to form the ring, it became dispersed and no septum was formed. Instead, some of the remaining cables reassembled at some distance to give rise to a normal septum (Fig. 4B; Movie S6).

### Recruitment of Actin-binding Proteins during CAR Formation

**Figure 6:**
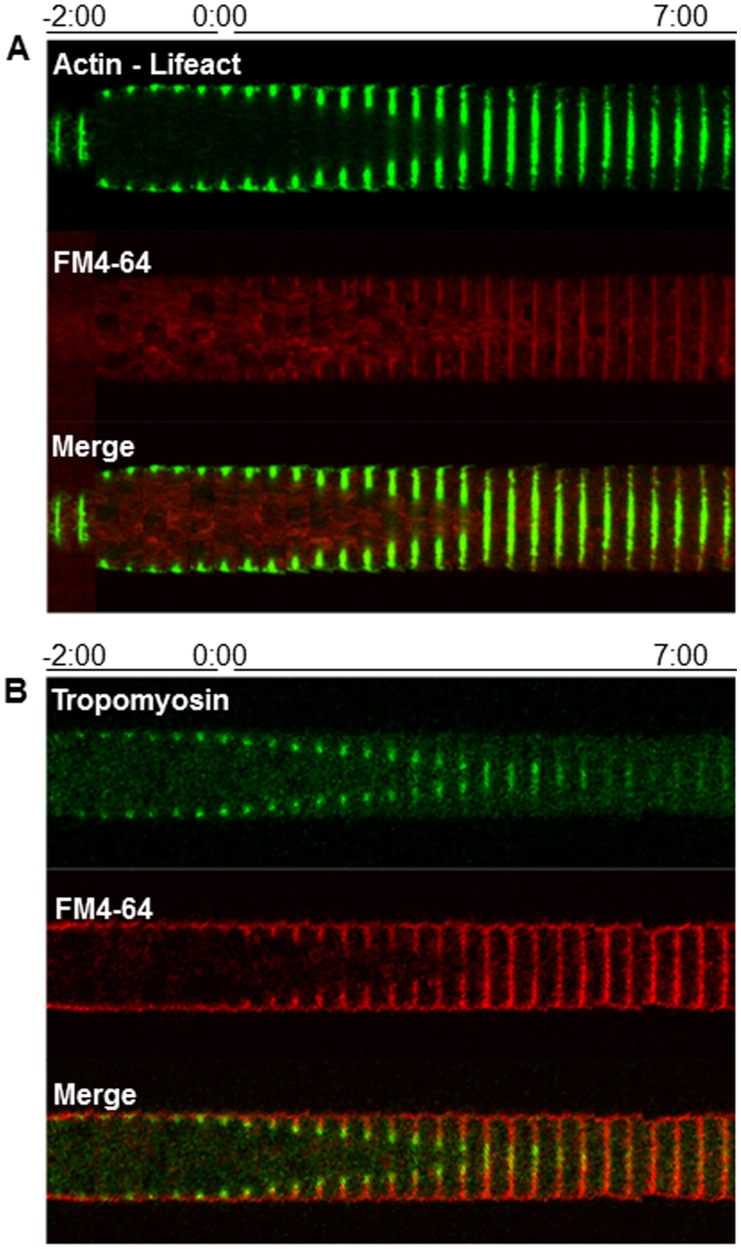
Chronology of the incorporation of actin (A) and tropomyosin (B) during septum development. Each kymographic series was constructed from medium plane images taken at 20(A) were made from the cortical plane. FM4-64 staining shows the boundaries of the developing septum. Time 0∶00 signals the earliest moment of membrane invagination.

**Figure 7:**
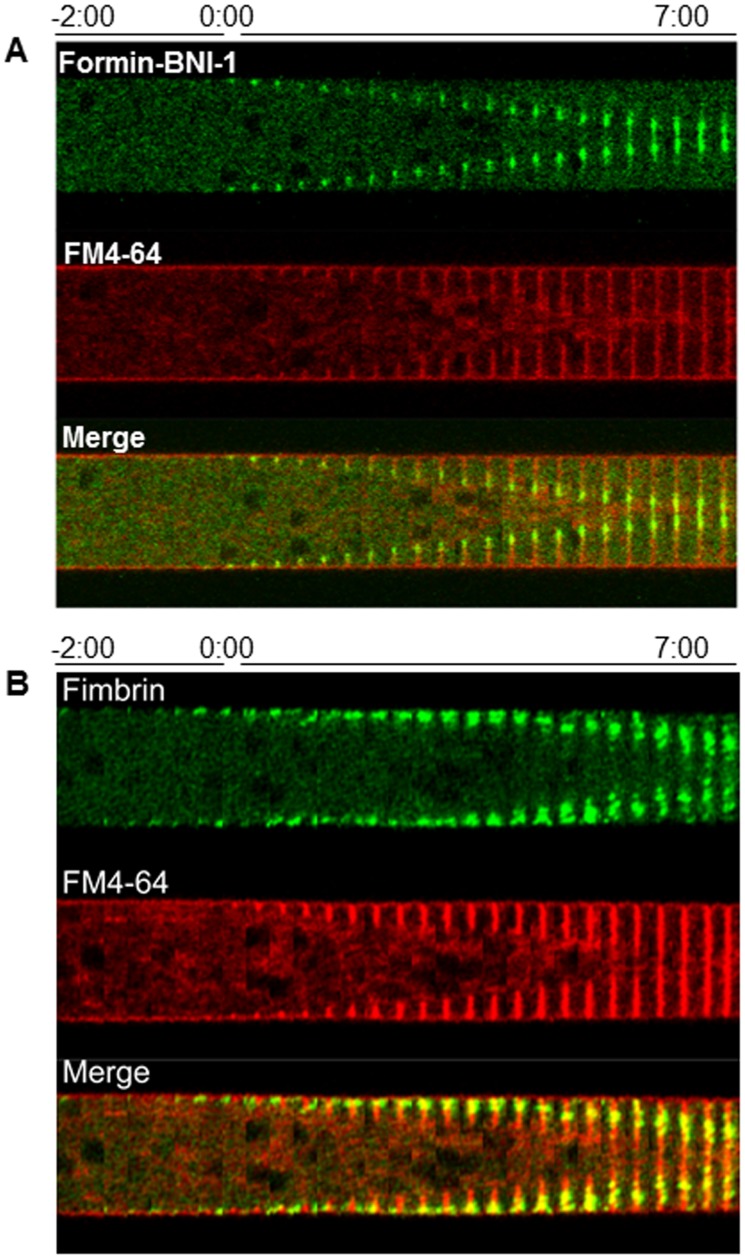
Chronology of the incorporation of Formin BNI-1 (A) and Fimbrin (B) during septum development. Each kymographic series was constructed from medium plane images taken at 20-64 staining shows the boundaries of the developing septum. Time 0∶00 signals the earliest moment of membrane invagination.

**Figure 8:**
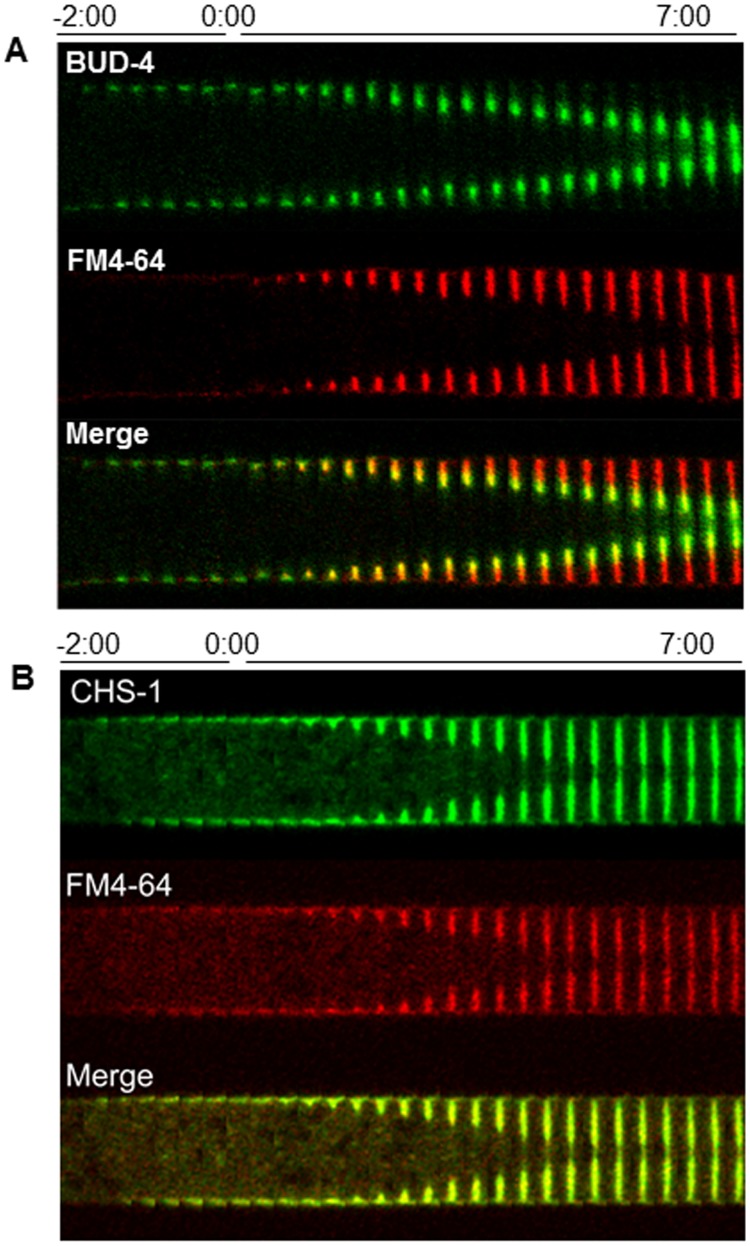
Chronology of the incorporation of BUD-4 (A) and CHS-1 (B) during septum development. Each kymographic series was constructed from medium plane images taken at 20-64 staining shows the boundaries of the developing septum. Time 0∶00 signals the earliest moment of membrane invagination.

The constricting actomyosin ring is a complex structure made of an actin skeleton anchoring other proteins (myosin, formin, and tropomyosin) that make it a functional constricting ring. To find out the order of assembly and precise location of these and other proteins, the Kymograph-like time series in Figures 6, 7 and 8 were constructed. The tagging of actin with Lifeact-GFP showed the CAR being established 2∶00 min before there was the first evidence of constriction (time 0∶00) (Figure 6A). Tropomyosin (TPM-1-GFP) appearance coincided with the initial stage of SAT assembly (Fig. 6B).

Formin (BNI-1-GFP) appeared as a cylindrical band of ∼10 µm in width at −3∶20 in the septation site (not shown). This band gradually coalesced into a sharp ring that reached its final width a few seconds before membrane ingrowth. The formin ring followed the advancing edge of the ingrowing membrane and was excluded from the rest of the septum plate. At the end of septation, the formin ring comprised the innermost 1/3 of the septum plate and then disappeared (Fig. 7A).

Fimbrin (FIM-GFP), a reporter for actin patches involved in endocytosis [35], accumulated around the septation site ∼10 seconds before membrane ingrowth. The actin patches flanked the developing septum on both sides (Fig. 7B). The actin binding proteins: tropomyosin, myosin, formin and fimbrin disappeared after septation had been completed.

### Recruitment of other Proteins

BUD-4 is an anillin-like protein that serves as a reporter of the BUD-4/BUD-3/RHO-4 complex [8]. BUD-4-GFP formed cortical spots in the vicinity of the future septation site two min before constriction of the CAR. A BUD-4 ring was formed at the leading edge of the ingrowing membrane. At the end of the septation process, BUD-4 covered the inner half of the septum and unlike other markers, it remained for the entire observation period (Fig. 8).

The genome of *N. crassa* encodes seven chitin synthases and all of them are present in the septa [16], [36]–[39]. For this study, we selected chitin synthase 1 (CHS-1) as a reporter of chitin synthase localization during septation. CHS-1 began to accumulate next to the CAR ∼2∶20 min (n = 13) before membrane ingrowth could be detected. CHS-1-GFP fluorescence disappeared from the cell surface prior to membrane ingrowth. Once membrane ingrowth started, chitin synthase accumulated in the growing edge of the developing septum in synchrony with its centripetal growth (Fig. 8B).

## Discussion

**Figure 9:**
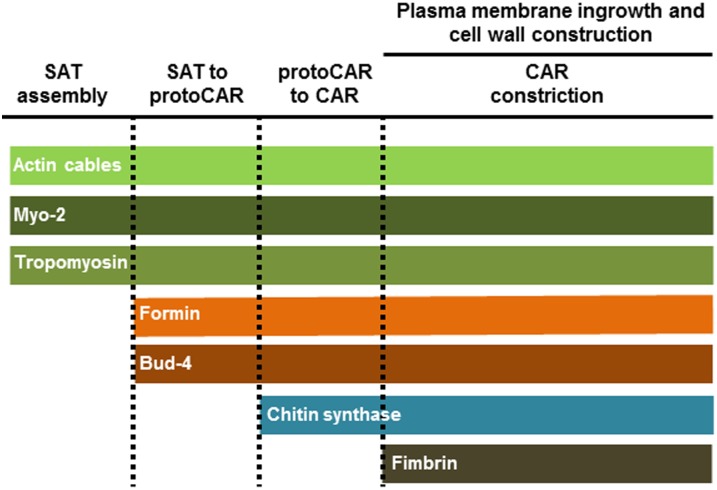
Schematic representation of the involvement of key proteins in different phases of septum development.

In recent years, interest in septum formation and its regulation in filamentous fungi has increased [15]–[16]. Septum formation is regulated in two stages: 1) the post-mitotic kinases of the SIN cascade and 2) the *in situ* process of cross wall construction. Many of the proteins involved in each stage of septation have been identified, and although the main mechanisms seem to be conserved between yeast and filamentous fungi [11]–[14], there are some important differences. There are several reports on specific components of the septation process in *A. nidulans* and *N. crassa*, [13]–[16], [21], [40]–[45]. Ours is a more comprehensive attempt to define the sequence of key events in septum formation in *N. crassa* mainly relative to actin and actin binding proteins (Fig. 9). We analyzed the chronology of the different proteins involved in septal development, including the homologs of markers that determine the septation site in other organisms (i.e. cell division plane). We used the actin reporter Lifeact-GFP [35], [46] as a *bona fide* marker of actin involvement throughout the entire process of septum formation.

The development of a septum at a defined position ca. 165 µm from the growing tip of mature hyphae, indicates the probable existence of a size-sensing mechanism for determining septation sites [18], [47]–[49]. Nuclear position and cell cycle are determinants for septum placement in *A. nidulans* (and possibly also *N. crassa*), although not every nuclear division triggers the formation of a septum [21], [25]. The SIN is essential for septum formation in yeasts and filamentous fungi [14], [50] and is thus a prime candidate for connecting cell cycle with septum placement. However, no evidence for a mechanistic involvement of the SIN in connecting both events currently exists in filamentous fungi [9]–[10], [51]–[52].

Other studies have suggested that post-mitotic proteins of the SIN cascade [14] are the first indicators of septation. However, in *N. crassa,* we have identified a tangle of thick actin cables (SAT) associated to tropomyosin and a class II myosin as the earliest signal of septum development. Although we did not perform co-expression experiments, it is safe to assume that all three components are simultaneously present in each filament of the complex.

A study of SIN components in *N. crassa*, showed that the kinase DBF-2, reaches the septation site around two min before plasma membrane ingrowth [9], [52], too late to be the trigger of SAT formation. Other candidates for triggering septum formation are the formin BNI-1 or the BUD-4/BUD-3/RHO-4 complex [8], [14], [52]. However, we found the SAT of *N. crassa* is formed and transformed into a contractile ring in the absence of formin BNI-1 or BUD-4. Instead, the timing of appearance of BUD-4 and formin BNI-1 suggests a critical role in the maturation of the proto-CAR into a CAR. Thus BUD-4 seems not to have a landmark function in septum formation in *N. crassa.*

A SAT seems to be formed partially from actin filaments or fragments that were left out in the transition from SAT to CAR from a preceding septation event. However, we cannot rule out the possibility that some of the actin filaments in the tangle arose de novo, as will be the case for the first septation site.

MYO-2 could provide the contractile force for SAT and CAR. MYO-2 appears in early stages of SAT formation. Tropomyosin is a protein that regulates the interaction between actin filaments and myosin [35], [53]–[54]. We showed it associated with the early stages of SAT formation, and it seems to remain as a part of the actin and myosin filaments as they travel from one septum to the next.

The conversion of a SAT into a CAR in *N. crassa* resembles in part the dynamics of actin during septation in *S. pombe* [55]–[56]. An analogous process of SAT formation and transformation to CAR has been called the “search, capture, pull, and release” (SCPR) model (reviewed [55]). However, there are some differences in SAT formation between *S. pombe* and *N. crassa*. The first difference is the accumulation of the anillin-like Mid1 nodes in the division site almost one hour before mitosis in *S. pombe* [27], [57]–[58]. Close to the beginning of mitosis, Mid1 recruits a broad band of nodes containing a Class II myosin (Myo2), and its two light chains Cdc4 and Rlc1; formin (Cdc12), and tropomyosin (Cdc8) [55], [59]–[62]. In *N. crassa* there is no Mid1 homologue, MYO-2 appears before any sign of plasma membrane ingrowth and its appearance coincides with that of actin. However, the ultimate trigger for SAT assembly in *N. crassa* remains unknown.

We followed the incorporation of CHS-1, into the septation process as a representative of the exocytic events that deliver wall building enzymes to the septum. It should be noted that *N. crassa* contains not one but seven chitin synthases and all have been found to travel to the septation sites [36], [38]–[39]. Localization of multiple chitin synthases at septa has been also reported in other fungi [63]–[71].

The fusion of vesicles carrying cell wall precursors to the plasma membrane in the septation site produces a simultaneous growth of both (cell wall and plasma membrane). There is no plasma membrane invagination *sensu stricto*; it is new membrane growing in a centripetal manner. Calculations on the amount of plasma membrane generated during apical cell wall growth indicate that an excess of membrane is produced that needs to be removed (Bartnicki-García, unpublished). The removal of the excess of plasma membrane generated during septation would probably depend on the same machinery that functions during endocytosis in other parts of the cell such as the endocytic subapical collar. A supposition supported by the finding of actin patches containing fimbrin, Arp2/3 complex and coronin; proteins associated with endocytic function [35], [72].

## Supporting Information

Movie S1 **Septum development in hyphae of** ***Neurospora crassa*** **visualized by fluorescent tagging of actin with Lifeact-GFP.** We can observe the growth of primary hyphae and the presence of actin in the Spitzenkörper, the subapical ring and the septation site.
(AVI)

Movie S2 **Dynamics of SAT formation and its transition to CAR. Actin labeled with Lifeact-GFP.**
(MPG)

Movie S3 **3D reconstruction of a z-stack of 0.9 µm confocal sections of a SAT.** Actin labeled with Lifeact-GFP.
(AVI)

Movie S4 **Dynamics of Lifeact-GFP labeled filaments during SAT and CAR assembly.** Fluorescent filaments derived from a recently formed septum moved towards (arrows) the next septation to form a new septum.
(AVI)

Movie S5 **Dynamics of MYO-2-GFP filaments during SAT and CAR assembly.** MYO-2 filaments derive from a recently formed septum and move towards (arrows) the next septation site in the leading hypha and also to the branch.
(AVI)

Movie S6 **Details of an instance of CAR abortion.** Actin labeled with Lifeact-GFP.
(AVI)
